# Defining the Content of an Online Sexual Health Intervention: The MenSS Website

**DOI:** 10.2196/resprot.4316

**Published:** 2015-07-03

**Authors:** Rosie Webster, Makeda Gerressu, Susan Michie, Claudia Estcourt, Jane Anderson, Chee Siang Ang, Elizabeth Murray, Greta Rait, Judith Stephenson, Julia V Bailey, The MenSS Trial Group

**Affiliations:** ^1^ eHealth Unit Research Department of Primary Care and Population Health University College London London United Kingdom; ^2^ Department of Infection and Population Health University College London London United Kingdom; ^3^ Research Department of Clinical, Educational, and Health Psychology University College London London United Kingdom; ^4^ BICMS, Barts and The London School of Medicine & Dentistry Barts Sexual Health Centre Queen Mary University of London, St Bartholomew’s Hospital London United Kingdom; ^5^ Homerton Sexual Health Services Homerton Teaching Hospitals London United Kingdom; ^6^ Engineering and Digital Arts University of Kent Kent United Kingdom; ^7^ PRIMENT Clinical Trials Unit Research Department of Primary Care and Population Health University College London London United Kingdom; ^8^ Department of Reproductive Health Institute for Women’s Health University College London London United Kingdom

**Keywords:** eHealth, behavior change, sexual health, condom use, sex education, heterosexual men, web-based intervention

## Abstract

**Background:**

Health promotion and risk reduction are essential components of sexual health care. However, it can be difficult to prioritize these within busy clinical services. Digital interventions may provide a new method for supporting these.

**Objective:**

The MenSS (Men’s Safer Sex) website is an interactive digital intervention developed by a multidisciplinary team, which aims to improve condom use in men who have sex with women (MSW). This paper describes the content of this intervention, and the rationale for it.

**Methods:**

Content was informed by a literature review regarding men’s barriers to condom use, workshops with experts in sexual health and technology (N=16) and interviews with men in sexual health clinics (N=20). Data from these sources were analyzed thematically, and synthesized using the Behavior Change Wheel framework.

**Results:**

The MenSS intervention is a website optimized for delivery via tablet computer within a clinic waiting room setting. Key targets identified were condom use skills, beliefs about pleasure and knowledge about risk. Content was developed using behavior change techniques, and interactive website features provided feedback tailored for individual users.

**Conclusions:**

This paper provides a detailed description of an evidence-based interactive digital intervention for sexual health, including how behavior change techniques were translated into practice within the design of the MenSS website. Triangulation between a targeted literature review, expert workshops, and interviews with men ensured that a range of potential influences on condom use were captured.

## Introduction

### Background

Sexually transmitted infections (STI) are a major public health problem, with high social and economic costs [1]. Diagnoses in England increased by 5% between 2011 and 2012, rising to 450,000 annual diagnoses of STI in 2013 [2]. Condoms are effective for prevention of STI; however, there are many barriers to successful use, for example decrease in sensation, interruption of sex, incorrect size or fit, or use of alcohol/recreational drugs [3,4]. Men have more power to influence use (given that it is them who wears the condom), so risk reduction and prevention efforts should be targeted at this group [3]. While there are many interventions aimed at improving sexual health for men who have sex with men (MSM), interventions specifically aimed at men who have sex with women (MSW) are lacking [5,6]. MSW report much less consistent condom use than MSM [7]; furthermore, men may be reluctant to discuss their sexual health with health professionals, partners or friends [8]. An interactive digital intervention may address this unmet need.

### Interactive Digital Interventions for Sexual Health

Interactive digital interventions are computer-based programs that provide information and one or more of: decision support, behavior change support, or emotional support for health issues’ [9]. Interactive digital interventions offer personally relevant, tailored material and feedback. Delivery via the Web and mobile devices offers private, anonymous, convenient access [10,11], which is particularly advantageous for sexual health content. Interactive digital interventions can potentially save clinic staff time as they require minimal delivery and training time compared to one-to-one structured discussions with patients, which is the current practice recommended in sexual health clinic settings [12]. Interactive digital interventions have been shown to have a moderate impact on condom use (*d*=0.259; 95% CI 0.201 - 0.317) [13], as well as increasing knowledge, self-efficacy and safer sex intention [9,13,14].

### Behavior Change Theory

Interventions that make more extensive use of theory and involve a higher level of user involvement in development tend to be more effective [15,16]. Such interventions tend to be complex; if we are to learn about “what works”, it is essential that such interventions are reported in enough detail to allow replication, implementation, and exploration of the mechanisms of action of an intervention [17]. A method developed for this purpose is to specify intervention content in terms of behavior change techniques (BCTs). BCTs are active components of an intervention designed to change behavior [18], and are applicable to a range of health behaviors [17]. A comprehensive theoretical framework which guides the intervention development process and suggests appropriate behavior change techniques (BCTs) is the Behavior Change Wheel (BCW) [19]. Intervention designers using this approach can select BCTs, considering the appropriateness for the population, setting, and intervention format. Despite using an established development framework, a creative leap is still needed to actually make an engaging, relevant intervention. User input is key to this process.

The Men’s Safer Sex (MenSS) website aimed to increase condom use in MSW, and was designed following extensive fieldwork with service users, using the Behavior Change Wheel to guide the development process and select appropriate BCTs (for details regarding the development process, see Webster and Bailey [20]). The website was designed to be viewed on a tablet computer in the clinic waiting room, thus utilizing the time that patients are waiting to be seen. This paper describes the content of the MenSS website, and the rationale for it.

## Methods

### Procedure

**Figure 1:**
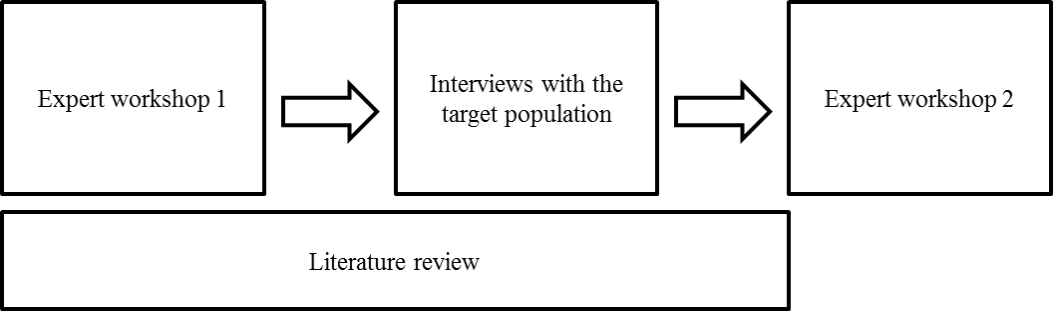
Flow diagram of data collection processes.

Three sources of information were used to determine the intervention content, format, and style (see Figure 1): research literature, expert views, and interviews with the target population (men in sexual health clinics). This evidence was discussed and evaluated in two expert consultation workshops. The development process was iterative, seeking comment and refinement of prototypes of website content from service users. The intervention took the form of an interactive website, rather than a mobile phone app, due to issues of privacy (ie an app would need to be stored on a user’s phone, which could be accessed by other people) and availability (ie not all users may own a mobile phone which could run an app).

Ethical approval was provided by the London – City and East NHS Research Ethics Committee (Reference number 13/LO/1801).

### Research Literature

A targeted literature review identified research on men’s barriers to condom use. Search terms included “men”, “heterosexual”, “condom”, and “barriers”. Databases searched included Web of Knowledge databases (including MEDLINE, EMBASE, and PsycINFO) and Google Scholar; selecting articles on risk factors for condom nonuse, theoretical correlates of condom use, and men’s barriers to using condoms. The full text of 27 papers was included, consisting of reviews, qualitative studies, and cross-sectional, longitudinal, population based, and experimental designs [21,22,23,24,25,26]. The findings of these papers were summarized and synthesized into themes and subthemes (see Table 1).

**Table 1:** Barriers and facilitators for condom use identified in the literature.

Theme	Subtheme
Barriers to condom use	Reduced pleasure or sensation when condoms are used
	Condoms cause interruption of sexual activity
	Condoms reduce intimacy
	Judging the risk of STI using appearance or behavior
	Saying one thing and doing another – the intention-behavior gap
	Partner perceptions/influence
	Difficulty using condoms
	Having sex under the influence of alcohol
	Low perceived susceptibility to STIs
	Condom problems (e.g., breaking, discomfort)
	Lack of awareness about risk of oral sex
Facilitators to condom use	Condoms as prevention against pregnancy
	Reflection on past behavior as a motivator
	Awareness/close personal experience of pregnancy or STI
	Seeing condom use as an “essential behavior”
	Desire to avoid STI
	Dislike of visiting clinics
	Having condoms available
	Communication about condoms with partner
Theoretical/psychosocial predictors identified in quantitative studies	Norms surrounding condom use
	Attitudes towards condom use
	Self-efficacy about using condoms
	Perceived susceptibility/risk
	Perceived benefits of and barriers to using condoms
	Outcome expectancies

### Expert Consultation

Two expert workshops were held to inform decisions, and to refine the focus, form and content of the intervention.

Attendees at the first (one day) workshop included 13 experts in the area of men’s sexual health and/or behavior change, including sexual health clinicians, health advisors, researchers, academic professors, and technology experts. The workshop was facilitated by RW and JB. Participants were asked to select the most important barriers and facilitators to condom use, and potential approaches to changing behavior. Participants were asked to work in small groups (2-5 people) to discuss tasks, and then give feedback to the whole group, which was audio recorded. Participants were also asked to write down their own personal views on individual worksheets.

A second (half day) workshop was held to guide final decisions regarding the intervention design and content and to inform the creative process of designing intervention features. This workshop included five experts in the fields of sexual health, sex education, and Web development (two of whom also attended the first workshop). Informed by the findings from the interviews with male clinic attendees, participants were asked to prioritize potential intervention content, and discuss the potential intervention features.

### Interviews With the Target Population

Semistructured qualitative interviews were conducted with 20 men who visited sexual health clinics, to gain information regarding barriers to and facilitators of condom use, potential intervention design, content, and mode of delivery.

Participants were recruited from two sexual health clinics in central London. Men attending sexual health drop-in clinics between February and April 2013, who were aged over 18 and had not been diagnosed with HIV or hepatitis, were eligible to participate. They were given a leaflet about the study and asked to approach the researcher if they wished to take part. Participants were aged between 20 and 52 (mean 31, SD 10.08); 7 identified as White British, 9 as Black (Black African or British), 2 as European, 1 as Chinese, and 1 as mixed ethnicity; 17 interviewees were currently sexually active with female partners, and 3 with male partners. The decision to focus solely on men who have sex with women (MSW) was made partway through the fieldwork process, hence a small number of men who have sex with men (MSM) were included in the sample. There was considerable overlap between MSM and MSW regarding the most prominent determinants of condom use. Some determinants were specific to MSM (e.g. a greater concern about contracting HIV), and so these were disregarded when synthesizing evidence.

Participants were asked about their experiences and views on using condoms and their interest in a potential sexual health website. Interviews lasted between 30 and 60 minutes, and were audio recorded. The recordings were listened to, initial themes summarized, and then analyzed using qualitative thematic content analysis [27], allowing inductive themes to emerge and using categories provided by the BCW to organize them [19,28].

## Results

### Overview

The development process of the intervention content, with reference to behavior change theory, is described elsewhere [20]. Here we describe the content and functionality of the intervention website, by providing each intervention topic, the rationale for it, the relevant behavior change techniques and the subsequent content included in the intervention.

### Barriers to Condom Use

#### Rationale

The interviews and literature review identified a multitude of potential barriers to condom use, and these barriers varied between individuals. Such barriers must be addressed in order to instigate behavior change; however, overwhelming all users with all content addressing all barriers may be off-putting. Interventions which are tailored to users are more likely to be effective [10].

#### BCTs

The relevant BCT was problem solving.

#### Content

**Figure 2:**
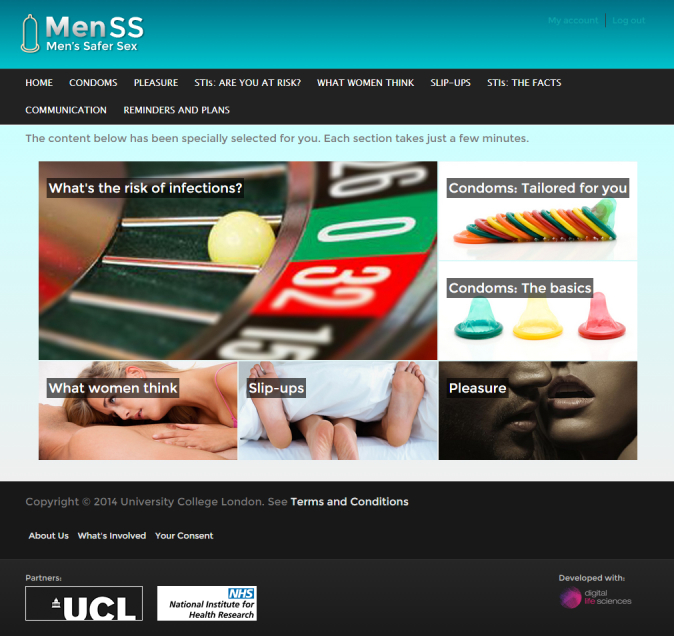
MenSS homepage with carousel of personalised tailored content.

On first using the website users were asked to select the reasons why they personally did not use condoms from 12 possible options, which were identified through the fieldwork (condoms too tight or uncomfortable; reduced pleasure; not knowing when or how to suggest it; being drunk or having taken drugs; losing erection; being in a relationship; difficulty stopping in the heat of the moment; partner not wanting to use condoms; partner might be offended; sex doesn’t feel as good; STIs are easily treated; they often break or slip off). The homepage (see Figure 2) was then tailored to each individual user by ensuring that the content that addressed the barriers selected was displayed prominently in the centre of the page (although all users could access all content through the global navigation bar).

### Condoms: The Basics

#### Rationale

Although data from the workshops and interviews suggested that men feel competent in applying condoms, the literature review identified high rates of errors and problems in condom use [29,30].

#### BCTs

Relevant BCTs included instruction on how to perform the behavior and demonstration of the behavior.

#### Content

**Figure 3:**
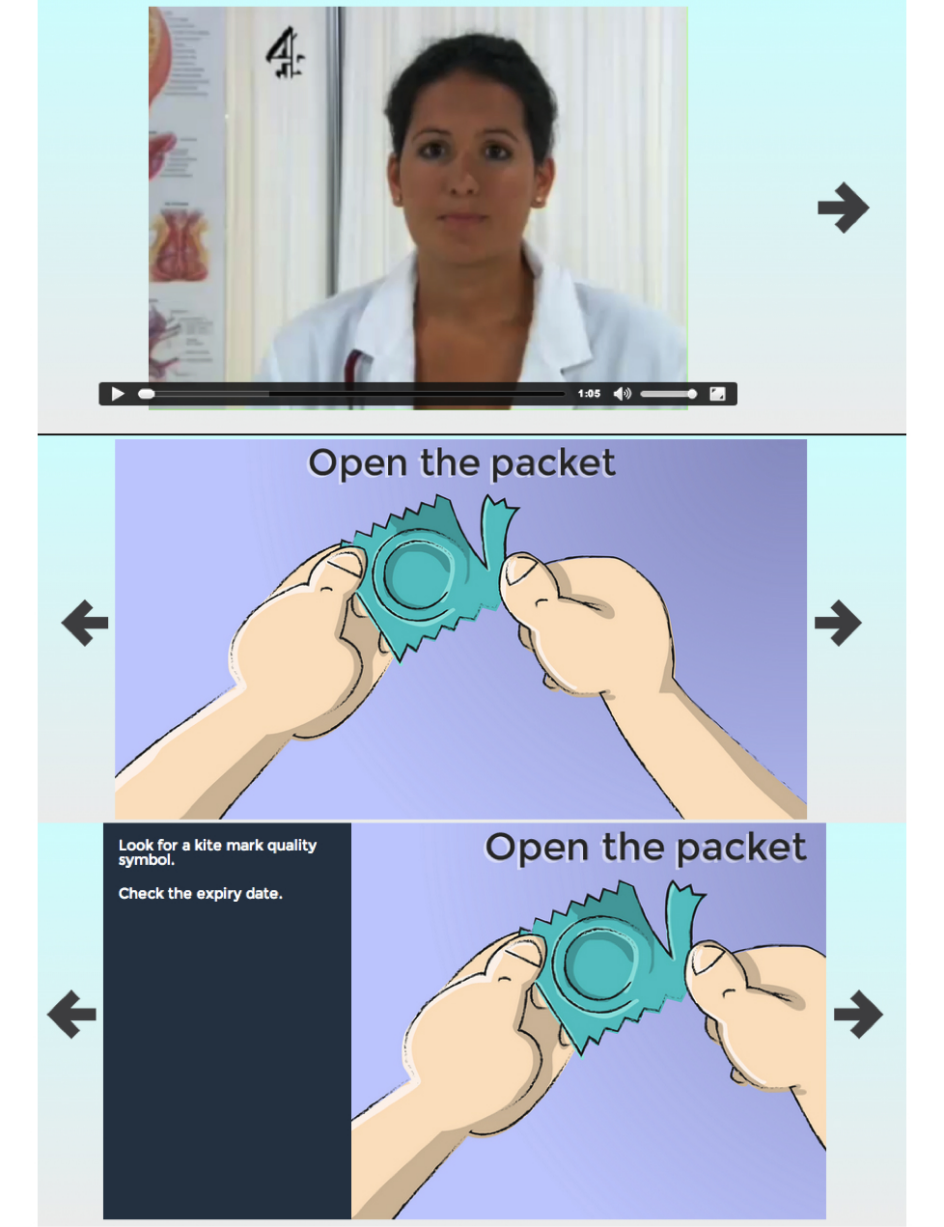
Condoms: The Basics – skills in correct condom application.

This section included a short video demonstration and a click-through slide show, which provided advice about using condoms correctly, highlighting the key steps in condom use and areas where people often make mistakes (see Figure 3).

### Condoms: Tailored for You

#### Rationale

Evidence from our qualitative work and the literature suggested that condom size and type impact strongly on acceptability of condoms, with poorly fitting or thicker condoms being viewed more negatively. Incorrect condom size was also related to problems such as breakage [31].

#### BCTs

The relevant BCT was problem solving.

#### Content

**Figure 4:**
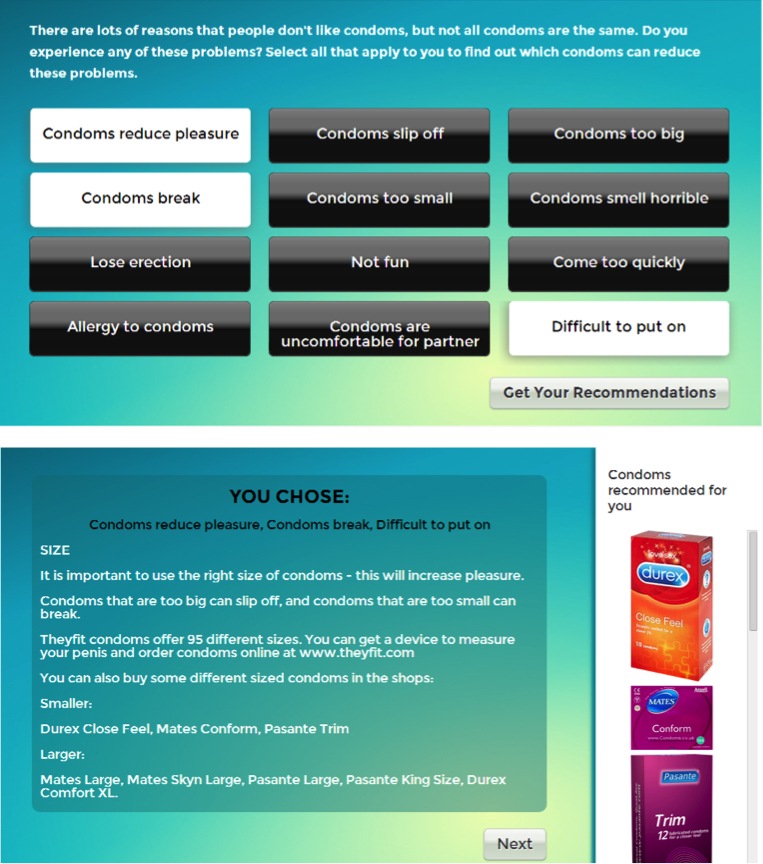
Condoms: Tailored for you – tailored feedback on selected barriers to condom use.

The intervention website aimed to educate men about different sizes and types of condom, using a tailored feedback activity. In this activity, users were asked to identify problems they had with condoms, and then offered tailored advice about and recommendations for condom types to help address those problems (see Figure 4). For example, men suggesting that condoms were uncomfortable, small, or problematic due to breaking were offered advice about larger types of condoms. The format of this activity was similar to the “Barriers to condom use” activity (above); but focused on problems with the actual condom (rather than problems surrounding condom use in general), and gives specific condom-related feedback and recommendations.

### Pleasure

#### Rationale

All data sources indicated that the belief that condoms reduce the pleasure of sex is a very important and common barrier to condom use.

#### BCTs

Relevant BCTs included the use of nonspecific incentive, restructuring the physical environment, instructions on how to perform the behavior, behavior substitution, information about health consequences, focus on past success, distraction, behavioral practice/rehearsal, anticipated regret, information about social and environmental consequences, and social incentive.

#### Content

This section incorporated written advice and videos. It gave advice about how to improve pleasure with condoms, how sex with condoms might be preferable to sex without condoms, for example by reducing worry (nonspecific incentive), how there are types of condom that may be more pleasurable, and how to enjoy nonpenetrative sex (behavioral substitution). BCTs were conceptualized within written text; for example, the “anticipated regret” technique encouraged users to focus on avoiding the worry and hassle that may follow an episode of unprotected sex; the “focus on past success” technique was incorporated by encouraging men who had previously had problems with loss of erection to focus on occasions when they had not lost their erection.

### STIs: Are You at Risk?

#### Rationale

All the data sources suggested that men were aware of the benefits of using condoms and of some of the risks of unprotected sex [21]. However, our interviews highlighted a number of widespread incorrect beliefs about the risk of STIs (e.g. partners who are known to them or “seem clean” are viewed as less risky).

#### BCTs

Relevant BCTs included receiving information about health consequences and the experience of vicarious consequences.

#### Content

**Figure 5:**
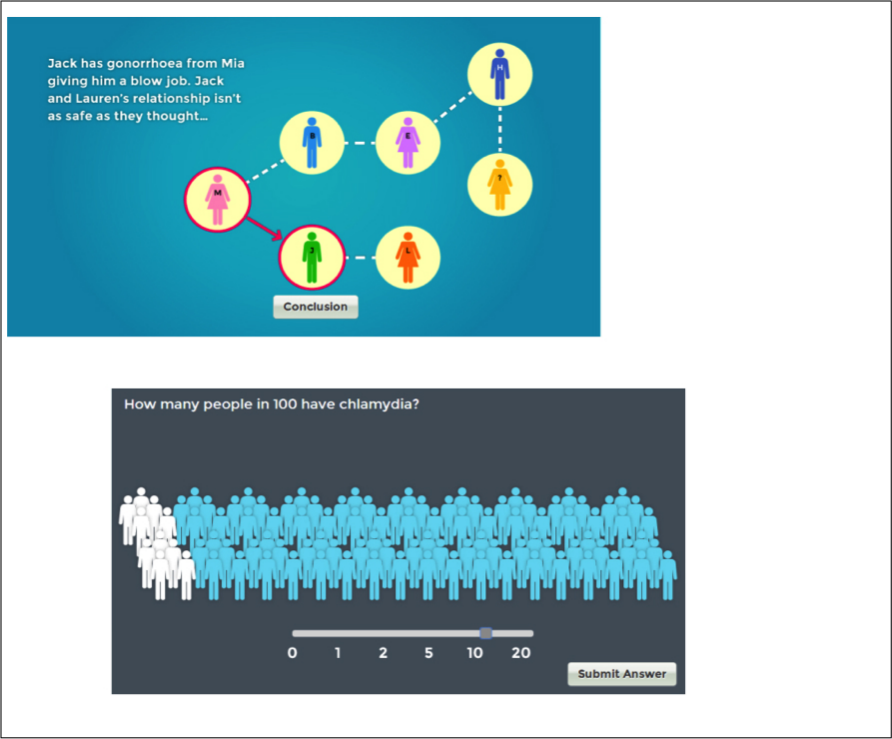
STIs: Are you at risk? – infographics illustrating potential risks of STI.

The intervention included two interactive activities addressing STI risk, emphasizing that risk levels cannot be judged (see Figure 5). First, in “What’s the risk of STIs?” a quiz presented facts and figures regarding STIs and their transmission (e.g. the number of people with undiagnosed HIV). This conceptualized the BCT of “information about health consequences” in an interactive and visually appealing manner. Second, in “Are relationships safe?” two animated diagrams demonstrated the way that STIs may spread within a network, common methods of transmission that people may not be aware of (e.g. oral sex), and how relationships may not be “safe”. This activity encompassed the BCT of “information about health consequences”, and also used “vicarious consequences”, by demonstrating the impact of risky sexual behaviors on others.

### What Women Think

#### Rationale

Both our interviews with men and the literature [24] identified fear of partners reacting negatively to suggestions of condom use as an important barrier. The literature also suggests that self-concept and personal values are related to carrying and using condoms [24,32]; if a healthy behavior is consistent with one’s identity, one may be more likely to perform the behavior [33]. Our workshops and interviews identified fostering a sense of responsibility towards others as a potentially important factor in condom use.

#### BCTs

BCTs considered relevant included receiving information regarding others’ approval and social incentive.

#### Content

This section included articles and videos portraying women as approving of men taking the responsibility for condom use and women not being offended by the suggestion of condoms. It also provided advice on responding to women who appeared to be offended. BCTs were conceptualized in written text and in videos; for example, “social incentive” was offered by suggesting that women would view men positively if they suggested condom use.

### Slip-Ups

#### Rationale

Pleasure or lust (being caught “in the heat of the moment”) was a widely quoted reason for non-use within our interviews with men and in the literature. All data sources identified alcohol as a strong barrier to condom use. The importance of carrying/availability of condoms has been related to condom use in the literature, and lack of availability was identified as a barrier by experts in the workshops.

#### BCTs

A wide number of BCTs were used here, including problem solving, verbal persuasion about capability, information about health consequences, instruction on how to perform a behavior, information about antecedents, restructuring the physical environment, anticipated regret, mental rehearsal of successful performance, information about social and environmental consequences, and nonspecific incentive.

#### Content

This section included articles and videos giving specific advice about how to overcome barriers due to the “heat of the moment” and intoxication by discussing condoms with a partner in advance (instruction on how to perform the behavior), considering potential regret (anticipated regret), and avoiding sex when under the influence of alcohol (information about antecedents). It also provided advice regarding making condoms available (e.g., carrying them, having them near the bed) (restructuring the physical environment). Again, BCTs were conceptualized in written text; for example, the “verbal persuasion about capability” technique included messages that men would be able to use condoms, despite high levels of arousal or intoxication. The “nonspecific incentive” technique was incorporated by telling users that if they wait to have sex when they are not intoxicated, they may perform better, please their partner more, and get a better reputation for being a good lover.

### STIs: The Facts

#### Rationale

In the qualitative interviews, men showed a lack of concern for catching STIs, as they did not feel that they had substantial negative consequences for men.

#### BCTs

The relevant BCT in this case was information about health consequences.

#### Content

Users were presented with common misconceptions or questions about STIs and their transmission (e.g. “STIs are easily treated, aren’t they?”), which could be clicked to reveal the answer and some brief information.

### Communication

#### Rationale

While the literature [21] and the experts in our workshop suggested that difficulties in negotiating condom use were a more salient barrier for women, evidence from the interviews with men suggested that for some this was an issue, and for most men the opinions of their partner were important when deciding whether to use a condom or not.

#### BCTs

Relevant BCTs included instruction on how to perform the behavior, information about social and environmental consequences, information about others’ approval, information about health consequences, and verbal persuasion about capability.

#### Content

This section offered information about specific strategies for suggesting, discussing, and negotiating condom use. BCTs were conceptualized in written information; for example, “information about social and environmental consequences” included giving advice that talking about condoms before sex would mean both partners can relax and enjoy it, rather than worrying about STIs or pregnancy; “information about others’ approval” included reassurance that most women would not be offended by the suggestion of condom use.

### Reminders and Plans

#### Rationale

Based on evidence from the literature [34,35] and our workshops, goal setting was identified as an important method of encouraging behavior change.

#### BCTs

Relevant BCTs were goal setting (behavior), action planning, and reviewing behavior goals.

#### Content

**Figure 6:**
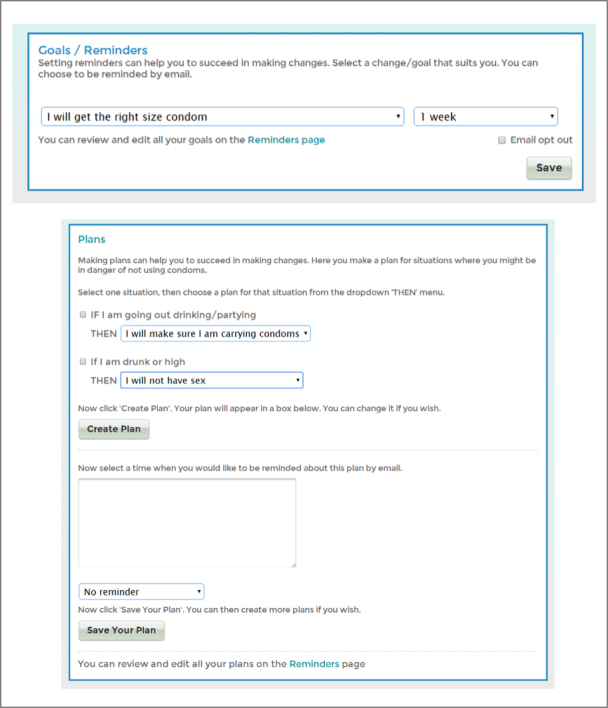
Reminders and plans feature.

In each section of the website, users were offered goals to set which related to the website content (see Figure 6). When selected, these goals populated users’ own personalized “Reminders and plans” page. Users could opt to receive a reminder by email at a specific time, set time-limited goals (e.g. “I will purchase my recommended condoms” by a selected date), or choose event-specific goals, by forming an implementation intention [36] (“if-then plan”), identifying a potential situation where condom use may be unlikely, and then selecting a response to that situation.

### Strategies for Engagement

#### Rationale

Whilst access to the website was provided at baseline in the clinic setting, the website was extremely comprehensive, and so most users would not have time to explore all content in one visit. Furthermore, the goal setting tasks were designed to support change over time, through users returning to the website to review their goals. It was therefore important to encourage repeated visits to the website. Increased engagement with an interactive digital intervention can lead to increased effectiveness [37]. Encouraging users to engage in interactive digital interventions, particularly over a long period of time, is notoriously difficult [38]. Email prompts can be used to increase engagement with the intervention [39].

#### BCTs

Relevant BCTs included the use of prompts and cues, information about health consequences, and reviewing behavior goals.

#### Content

Users were sent monthly emails, prompting them to visit the intervention website again, in the hope that they would explore content that they had not previously viewed. These engagement emails contained “teasers” regarding website information and links to the website (e.g. “Do you know how many people have Chlamydia? Find out here”). In addition, if users set goals or implementation intentions within the website, they could select the option of being reminded via email. These emails asked users if they had achieved their goal, and prompted them to return to the website to review their goals.

## Discussion

### Principal Findings

This paper provides a description of the content for an interactive digital intervention aimed at increasing condom use in men, and the rationale for it. Triangulation between a targeted literature review, expert workshops, and interviews with the target population, all led by a multidisciplinary team, ensured that a range of potential influences on condom use were captured and that feedback to men on barriers to condom use was relevant to them. The resulting intervention is extensive, tailored to individual needs, and targets a wide set of influences on sexual behavior. This is in line with recommendations that sexual health interventions should use a holistic approach to sexual health and well-being [40].

As with many complex interventions, the MenSS website contains multiple components, targeting a number of influences on behavior. It can therefore be difficult to determine which part(s) of the intervention are effective, via what mechanisms. Online interventions offer the possibility of easily monitoring patterns of intervention use, including its component parts, which can assist in the analysis of the mechanisms of action of an intervention. Clearly describing the intervention aims and content can assist in this analysis. BCTs provide a standardized method for this process of describing intervention content.

The use of standardized BCTs to specify the intervention content provided two advantages. First, the BCTs provided ideas for website features and health promotion messages (so the authors did not start with a “blank canvas”). Second, the BCTs were used to specify the content in standardized terms to facilitate replication, make judgments regarding quality, and allow comparisons with other interventions [18]. Translating BCTs into interactive website features can be a difficult process, requiring a certain level of creativity. Given that the content of complex interventions is often not described in detail [41], building a repository of examples of such features would be a valuable resource for intervention designers.

### Limitations

The study had some limitations. For example, while we collected detailed data to specify intervention content, the literature review was targeted, rather than fully systematic, due to time and resource constraints. This means some relevant evidence may have been missed. However, the inclusion of a number of systematic reviews within the literature review mitigates this concern. A second limitation is that all men interviewed during the development process were sampled from sexual health clinics within inner London, thus potentially limiting the transferability to other populations and settings. However, the findings from our literature review confirmed the importance of the emergent themes from the interviews.

### Conclusions and Future Work

This paper provides a detailed description of an evidence-based interactive digital intervention for sexual health, including how BCTs were translated into practice within the design of the MenSS website. It is hoped that this will assist intervention developers in their development work and reporting in terms of BCTs. A pilot study is currently underway to determine the feasibility of evaluating the intervention in a full scale randomized controlled trial.

## Abbreviations

BCT: behavior change technique
BCW: behavior change wheel
IDI: interactive digital intervention
MenSS: Men’s Safer Sex
MSM: men who have sex with men
MSW: men who have sex with women
STI: sexually transmitted infections
HIV: human immunodeficiency virus
